# Supplementary material for: A Combined Cyanine/Carbomer Gel Enhanced Photodynamic Antimicrobial Activity and Wound Healing
Source: Nanomaterials (Basel). 2022 Jun 24;12(13):2173. doi: 10.3390/nano12132173 (PMC9268119; doi:10.3390/nano12132173)
Supplement: Supplementary file 1 [file nanomaterials-12-02173-s001.zip › nanomaterials-1725986-supplementary.pdf]

nanomaterials

Supplementary Materials

A Combined Cyanine/Carbomer Gel Enhanced Photodynamic Antimicrobial Activity and Wound Healing

Ming Guan [1,†], Guangyu Chu [1,†], Jiale Jin [1], Can Liu [1], Linxiang Cheng [1], Yi Guo [2], Zexing Deng [3,*] and Yue Wang [1,*]

1 Spine Lab, Department of Orthopedic Surgery, The First Affiliated Hospital, Zhejiang University School of Medicine, Hangzhou 310003, China; guanm@zju.edu.cn (M.G.); 12118297@zju.edu.cn (G.C.); 22018143@zju.edu.cn (J.J.); 12018499@zju.edu.cn (C.L.); q773916513@zju.edu.cn (L.C.)
2 Shaanxi Key Laboratory of Brain Disorders, Xi'an Medical University, Xi'an 710021, China; guoyi@xiyi.edu.cn
3 College of Materials Science and Engineering, Xi'an University of Science and Technology, Xi'an 710054, China
* Correspondence: biomaterial@xust.edu.cn (Z.D.); wangyuespine@zju.edu.cn (Y.W.); Tel.: +86-136-7918-7589 (Z.D.); +86-0571-8723-6128 (Y.W.)
† These authors contributed equally to this work.

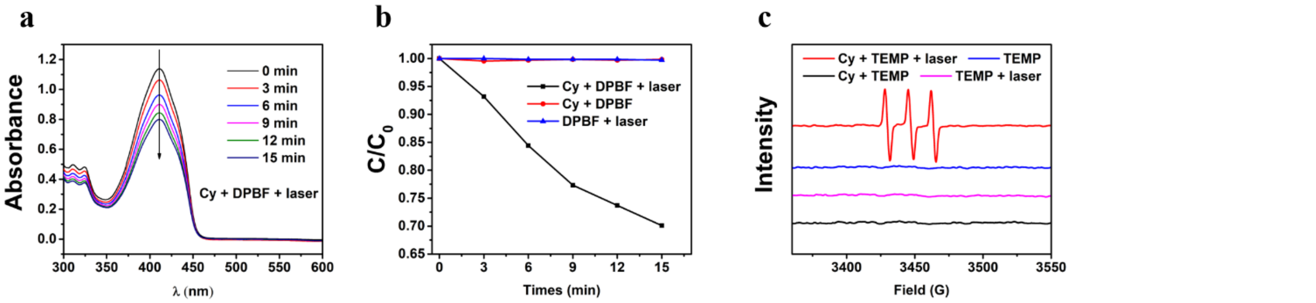

Figure S1. The generation of singlet oxygen ($^1O_2$) by photo-activated Cy. (**a**) Time-dependent UV-Vis absorption spectra of DPBF with Cy under laser irradiation. (**b**) Normalized absorbance of DPBF in different conditions. (**c**) EPR spectra of Cy in the presence of TEMP in different conditions.

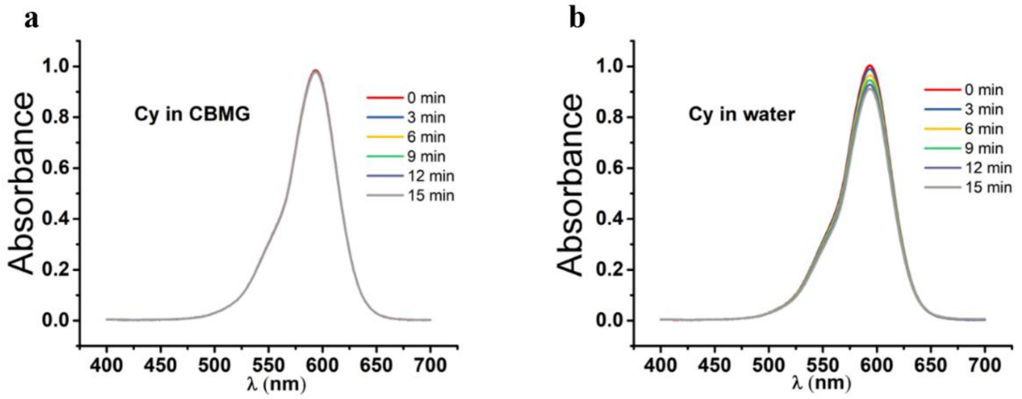

Figure S2. Photostability of Cy in different media. (**a**) CBMG. (**b**) water.

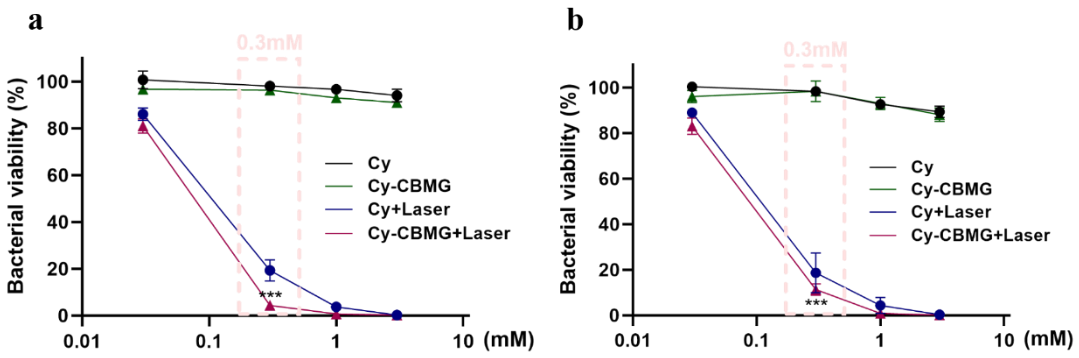

Figure S3. Bacterial viability after various concentration of Cy (0.03, 0.3, 1, 3 mM) in water or carbomer (laser −; laser +). (**a**). MRSA. (**b**). ESBL *E. coli*. ***: a statistically significant difference between groups, $p < 0.001$.

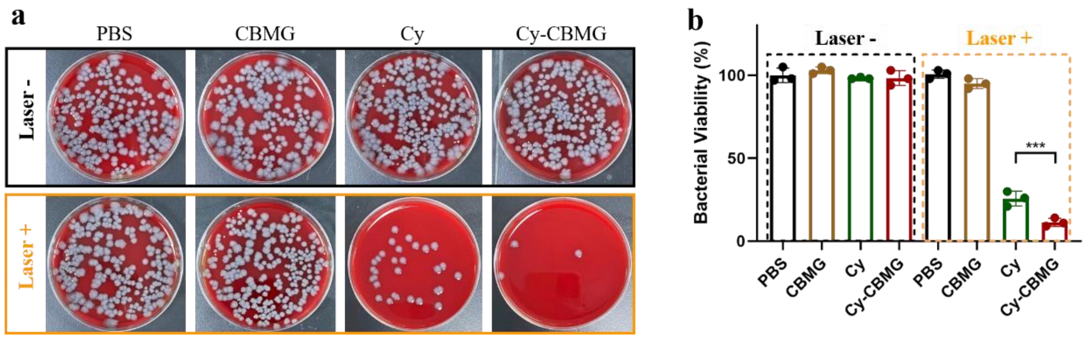

Figure S4. Antibacterial efficiency of samples towards ESBL *E. coli*. (**a**) CFUs of ESBL *E. coli* on TSA plate after treated with or without laser irradiation (600 nm, 100 mW/cm^2). (**b**) Bacterial viability analysis based on CFUs. ***: a statistically significant difference between groups, $p < 0.001$.

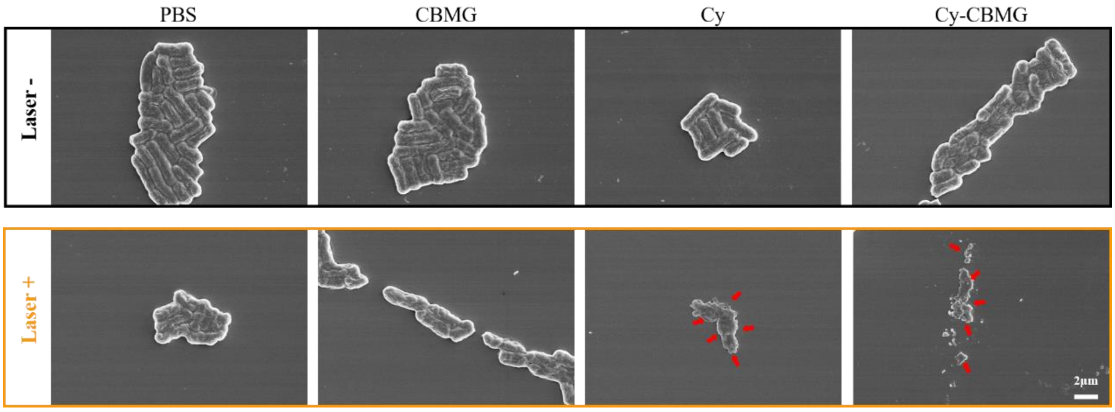

Figure S5. SEM images of ESBL *E. coli* after various treatments. Red arrows indicated bacteria with morphology changes.

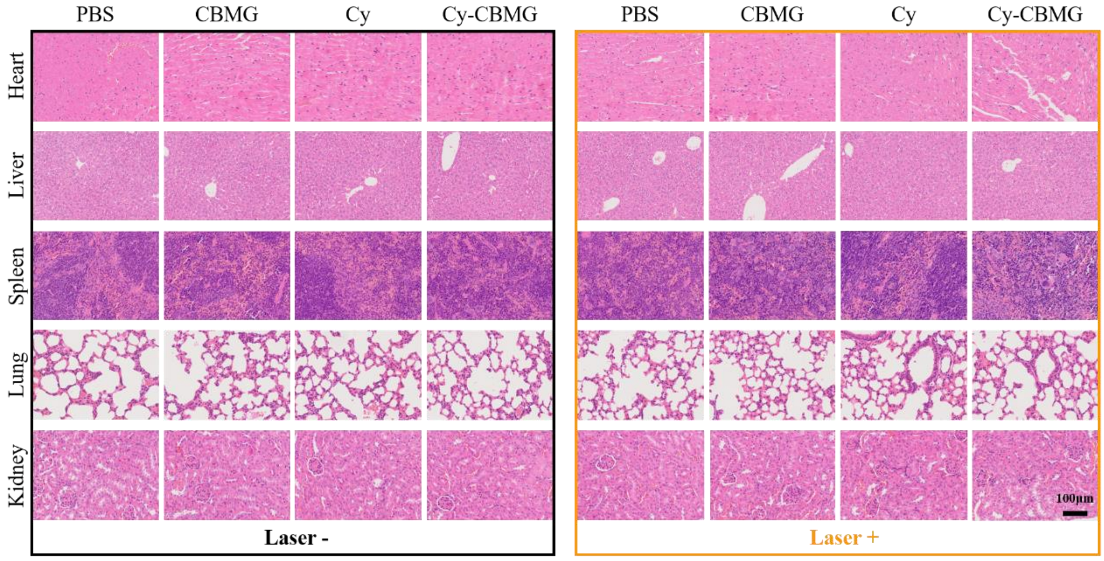

Figure S6. Biocompatibility evaluation of samples in vivo.
